# Fostering Flexibility in the New World of Work: A Model of Time-Spatial Job Crafting

**DOI:** 10.3389/fpsyg.2019.00505

**Published:** 2019-03-19

**Authors:** Christina Wessels, Michaéla C. Schippers, Sebastian Stegmann, Arnold B. Bakker, Peter J. van Baalen, Karin I. Proper

**Affiliations:** ^1^Department of Technology and Operations Management, Rotterdam School of Management, Erasmus University, Rotterdam, Netherlands; ^2^Institute of Psychology, Goethe University Frankfurt, Frankfurt, Germany; ^3^Center of Excellence for Positive Organizational Psychology, Erasmus University, Rotterdam, Netherlands; ^4^Faculty of Economics and Business, University of Amsterdam, Amsterdam, Netherlands; ^5^Centre for Nutrition, Prevention and Health Services, National Institute for Public Health and the Environment, Bilthoven, Netherlands

**Keywords:** flexible working practices, new world of work, job crafting, time-spatial job crafting, work engagement, person-job fit, time/spatial-demands fit, work-life balance

## Abstract

In today’s “new world of work,” knowledge workers are often given considerable flexibility regarding where and when to work (i.e., time-spatial flexibility) and this has become a popular approach to redesigning work. Whilst the adoption of such practices is mainly considered a top-down approach to work design, we argue that successful utilization of time-spatial flexibility requires proactivity on the part of the employee in the form of *time-spatial job crafting*. Previous research has demonstrated that time-spatial flexibility can have both positive and negative effects on well-being, performance, and work-life balance; yet remains mute about the underlying reasons for this and how employees can handle the given flexibility. Drawing on research from work design, we posit that in order for employees to stay well and productive in this context, they need to engage in time-spatial job crafting (i.e., a context-specific form of job crafting that entails reflection on time and place), which can be considered a future work skill. We propose a theoretical model of time-spatial job crafting in which we discuss its components, shed light on its antecedents, and explain how time-spatial job crafting is related to positive work outcomes through a time/spatial-demands fit.

## Introduction

*Where shall I work today? At home? In the office? Where in the office? In the silence area? In the open office area? When shall I start working? Before I bring the kids to school or afterward?* These are only some of the various questions knowledge workers are confronted with in the contemporary world of work every day. Commencing with advances in information and communication technologies (ICT), a new way of working emerged where knowledge work organizations have gradually moved from using traditional offices with permanent workplaces to adopting a more hybrid approach (e.g., Microsoft Netherlands). This enables knowledge workers such as academics, consultants or analysts to work from different work venues both outside the central office (e.g., a home office, a client’s premises, or on the go) and inside it (e.g., open office space, silent areas) (cf. Vos and van der Voordt, 2001) that are designed for the execution of particular tasks (e.g., collaborative work, focused work) (Becker and Steele, 1995). Along with the increased flexibility regarding where to work, employees also have greater flexibility regarding when to work. This implies that employees are better able to control and adjust their working hours to suit their private demands (Baltes et al., 1999). Flexible working times have become a relatively widespread policy within the European Union – especially in the Northern and Western member states (European Commission, 2010). Flexibility in terms of when and where to work is also known as time-spatial flexibility (Peters et al., 2009). Time flexibility is considered to be a supportive HR policy helping employees in knowledge work organizations to manage all the different work and private demands (European Commission, 2010).

However, prior research has shown equivocal and contradicting findings regarding the effects of time-spatial flexibility; it has been related to both negative (e.g., Brennan et al., 2002; Kelliher and Anderson, 2008) and positive (e.g., Gajendran and Harrison, 2007; Kelliher and Anderson, 2008; McElroy and Morrow, 2010) outcomes in terms of employee well-being, performance, and work-life balance (for reviews see De Croon et al., 2005; De Menezes and Kelliher, 2011).

Given these equivocal findings regarding the effectiveness of time-spatial flexibility, the question arises how employees can make informed choices regarding workplaces, work locations, and working hours to ensure well-being, high performance, and a good work-life balance on a daily level. Previous literature is relatively mute on why and when flexible work designs lead to positive or negative effects neglecting the role of possible mediators, moderators, and time in this relation (De Menezes and Kelliher, 2011). In the current paper, we respond to calls to come up with more sophisticated research models in this area. Since a flexible work design is a central element in the European employment strategy (European Commission, 2010) and a growing number of organizations implement (aspects of) time-spatial flexibility (Vos and van der Voordt, 2001; European Commission, 2010), it is imperative to know which strategies are most effective in dealing with increased flexibility.

**FIGURE 1:**
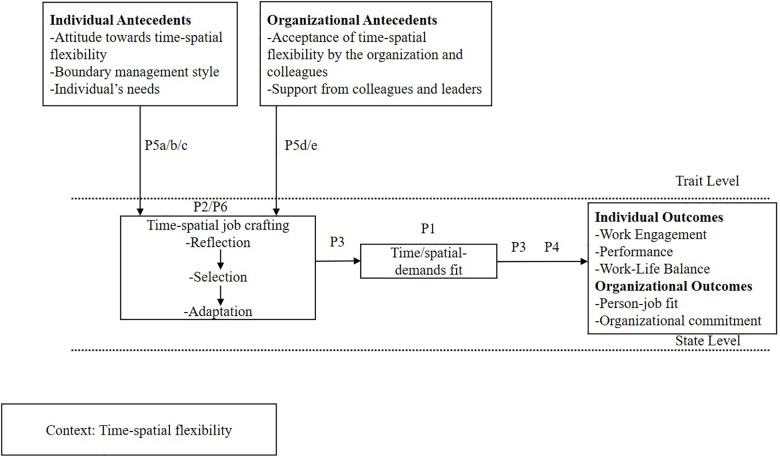
A model of time-spatial job crafting.

To address these challenges, we develop a theory and model of time-spatial job crafting, in which we propose that a large part of the negative outcomes of flexibility are likely due to a misfit between personal and task demands and working hours, work locations, and workplaces. Hence, a first thing we propose in Figure 1 is that employees in knowledge work organizations need to optimize a time/spatial-demands fit on a day-to-day basis. However, finding this fit seems to be particularly difficult given the mixed findings of flexibility. Therefore, a second thing we propose is that in order to find a time/spatial-demands fit, employees should ideally engage in time-spatial job crafting. This should help them to capitalize on flexibility on a day-to-day basis and is related to positive outcomes by means of a time/spatial-demands fit.

Part of the problem of finding a good fit seems to be that to date, flexible working practices have been understood mainly as a top-down approach to work design (cf. Humphrey et al., 2007). We argue that a bottom-up work design approach (Humphrey et al., 2007) is needed to provide an optimal fit between personal and task demands and work locations, workplaces, and working hours on a day-to-day-basis. Job crafting could be such a bottom up approach to job design. It has been defined as proactive behavior by employees aimed at making changes to job characteristics such as tasks and relationships (Wrzesniewski and Dutton, 2001) or job demands and job resources (Demerouti and Bakker, 2014).

However, research on job crafting has been relatively mute about how job crafting is related to time and spatial dimensions of work. In the context of time-spatial flexibility, we argue that it is imperative that employees make conscious decisions regarding the time and spatial dimensions of their work to optimize a time/spatial-demands fit (i.e., the best time and location/place to work on a given task and given personal demands). We introduce the term time-spatial job crafting as a form of self-regulatory behavior (Higgins, 1987). Time-spatial job crafting refers to the extent to which employees reflect on specific work tasks and private demands, actively select workplaces, work locations, and working hours, and then potentially adapt the place/location of work and working hours or tasks and private demands to ensure that these still fit to each other (i.e., optimizing time/spatial-demands fit).

A core premise of this article is that time-spatial job crafting enables knowledge workers to benefit from time-spatial flexibility on a day-to-day basis by optimizing a time/spatial-demands fit. The model of time-spatial job crafting is thus proposed as a context specific model as it only relates to knowledge work organizations that offer time spatial flexibility in Western societies. In the following, we review literature on time-spatial flexibility and outcomes; introduce time/spatial-demands fit as a specific kind of fit and embed it into existing P-E fit literature; explain the different components of time-spatial job crafting as well as its antecedents on the trait level and elaborate on how time-spatial job crafting is related to positive outcomes on the state level.

Our model (see Figure 1) is important from both a theoretical and practical standpoint. Theoretically, the model extends literature on job crafting (Wrzesniewski and Dutton, 2001; Bakker et al., 2014), reflexivity (Schippers et al., 2014) and flexible work arrangements (Hill et al., 2008). In particular, it incorporates the role of time into flexibility research to explain the mixed findings of cross-sectional research in this area. Our model also contributes to the work design literature by emphasizing the importance of bottom-up approaches of work design in the new world of work. This paper also has important practical implications, as time-spatial job crafting may be of particular interest for employees working under a flexibility policy and their organizations. In particular, the model offers important handles for knowledge workers and knowledge work organizations on how to deal with the given flexibility and raises HR managers’ awareness for the optimal usage of flexibility.

## Conceptualization of Time-Spatial Flexibility

Time-spatial flexibility within the new world of work describes the context in which knowledge work employees have the ability to decide when, where, and for how long to work on a daily basis (Hill et al., 2008). Employees, who have the freedom to determine when and how long they work, have scheduling or time flexibility. A common form of time flexibility is flextime, which gives employees the freedom and control to adjust working hours to their personal needs (Baltes et al., 1999). This not only includes scope to vary the start and end point of a working day but also the length of the workday can be adjusted. Spatial flexibility allows work tasks to be carried out away from the office (e.g., at home, at a client’s premises, in the train, or in a coffee shop), and working away from the central office location is often referred to as teleworking (Nilles, 1998). Previous definitions of spatial flexibility have failed to include the notion of increasing flexibility *inside* the office environment. With greater flexibility inside the office environment, work tasks can be accomplished from different workplaces within the central office that are often designed with a specific kind of task in mind (e.g., silent areas, open office areas, meeting rooms, or brainstorm rooms) (Becker and Steele, 1995). In the current paper, we therefore include this notion of flexibility in the definition of spatial flexibility.

Even if an organization offers flextime and flexplace options, this does not guarantee that employees recognize these as such or actually make use of them (Hill et al., 2001). It is therefore important to differentiate between the more formal time-spatial flexibility provided by the employer (e.g., as part of an HR policy) and the actual time-spatial flexibility experienced by employees on a day-to-day basis, which we will focus on. This combination of time and spatial flexibility influences how employees carry out their work and thus brings both opportunities and risks for individuals (Karlsson, 2007).

## Consequences of Time-Spatial Flexibility

Offering time-spatial flexibility is often said to help employees in knowledge work organizations in being able to handle work and non-work obligations in a more balanced manner (Allen and Shockley, 2009) and is regarded as one of the main policies to cope with demands from both work and life (Poelmans and Chenoy, 2008). As time-spatial flexibility gives employees greater control over scheduling their workdays, employees are able to allocate work, and non-work time more efficiently in a way that fits their needs thereby creating balance between work and home life. For instance, not having to commute to the office saves commuting time which can be spent otherwise (Hill et al., 2003). Despite its main goal regarding handling responsibilities from both work and home in a better way, there exists great inconsistency regarding the actual effectiveness of time-spatial flexibility practices for work-life balance (Allen and Shockley, 2009). While some studies reported increases in work-life balance due to decreases in work-family conflict (e.g., Hammer et al., 1997; Hill et al., 2003; Madsen, 2003; Gajendran and Harrison, 2007); other studies found decreases in work-life balance due to greater blurring of boundaries (Kurland and Bailey, 1999) or no significant relation (Aryee, 1992; Hill et al., 1998).

Time-spatial flexibility and the choices that individuals make also affects employee’s well-being and performance. In their literature review particularly on the influence of office concepts on health and performance, De Croon et al. (2005) identified that office concepts – such as open offices spaces and telework offices – can have positive as well as negative effects on performance and well-being. For instance, on the one hand, in an open office space with several workstations, employees oftentimes have direct eye contact with each other. Due to this proximity, employees can easily be distracted by their co-workers (McElroy and Morrow, 2010). This kind of interruption and disturbance is assumed to increase cognitive workload because employees need to stop regularly and then refocus on the task at hand. This can be an energy-draining activity, which, will lead to exhaustion and diminished work performance. On the other hand, De Croon et al. (2005) note that that time-spatial flexibility can also increase well-being and performance. For instance, Ten Brummelhuis et al. (2012) found in their study that once an employee has decision latitude in terms of responding to emails and phone calls, the general efficiency and effectiveness of communication increases, leading to more work engagement.

A systematic review by De Menezes and Kelliher (2011) on flexible work arrangements and performance-related outcomes also found that flexible working arrangements can be both beneficial and detrimental for employees and their organizations. They conclude that so far the evidence fails to provide a clear business case for flexible work arrangements, but that future research should take into account moderators, mediators, and the role of time. In our theorizing, we aim to (1) propose a theory that incorporates these, and (2) propose when and why flexible work arrangements are related to better employee outcomes. Below, we present our propositions.

## Propositions

### Time/Spatial-Demands Fit

In light of the health-promoting and health-impairing influences of time-spatial flexibility on work outcomes, we argue that individuals can make choices over workplaces, work locations, and working hours that enable them to either exploit the advantages we have outlined above or run the risk of being affected by the disadvantages. Thus, times-spatial flexibility is not a good or bad thing *per se*; whether it turns out favorably or unfavorably depends on how each individual uses the flexibility and the extent to which they manage to optimize the time/spatial-demands fit. We argue that a great deal of the negative outcomes may result from a misfit between working hours, work locations, and workplaces and task and private demands. This has largely been neglected in previous theorizing efforts for why flexibility does not lead to positive outcomes. As can be seen in Figure 1, a first thing we propose is that in order to remain productive, engaged and to keep a good work-life balance when faced with time-spatial flexibility on a daily level, employees should ideally optimize a time/spatial-demands fit. Analogs to the task-technology fit perspective (Goodhue, 1997), where workers optimize the fit between work tasks and technology and technology and their abilities, we define time/spatial-demands fit as the fit between work tasks and work locations, workplaces, and working hours on the one hand and private demands and work locations, workplaces, and working hours on the other hand. Time/spatial-demands fit is different from person-environment fit (P-E fit) as it is not concerned with a fit between the person and the environment. Person-environment fit is conceptualized either as the fit between an employee’s characteristics and the characteristics of an organization (P-O fit) or as the fit between an employee’s competencies such as personal needs and abilities and the requirements of the job (P-J fit) (Kristof-Brown et al., 2005). Thus, the nature of the P-E fit literature is concerned with the fit between the person and the environment (Chatman, 1989). However, we argue that time/spatial-demands fit does not deal with the fit of the person with the environment. Time/spatial-demands fit is concerned with the fit between perceptions of a HR policy (flexible working hours and work locations) and office design (workplaces) on the one hand and work demands and private demands on the other hand. We propose that employees who have time-spatial flexibility need to match task and private demands to designated places, locations, and to working hours. Taken together, optimal health, performance, and work-life balance will be ensured if employees manage to create an optimal time/spatial-demands fit in the context of time-spatial flexibility. Thus, we suggest.

#### Proposition 1

In the context of time-spatial flexibility, employees need to optimize a time/spatial-demands fit.

### How Can Employees Optimize Time/Spatial-Demands Fit? From Job Crafting to Time-Spatial Job Crafting

In order to optimize the time/spatial-demands fit, employees should ideally engage in what we term *time-spatial job crafting*. In the work design literature, job crafting is seen as a specific form of proactive behavior and shares distinct features with it, such as initiative-taking behavior or anticipating a future situation and adapting behavior accordingly (Parker and Collins, 2010). The central tenet of current job crafting conceptualizations is that employees alter aspects of their job of their own accord. Originally, job crafting has been defined in terms of physical and cognitive changes that employees make to the task or to their relationships at work (Wrzesniewski and Dutton, 2001). According to the latter authors, employees may modify three different aspects of their job – namely the task itself, their relationships with others, and/or their perception of the job (i.e., cognitive crafting). Recently, scholars extended the conceptualization of job crafting to also include self-initiated skill development (Lyons, 2008) and modifying job demands and resources (Tims et al., 2012). According to Tims et al.’s (2012) reasoning, employees proactively increase structural and social job resources, as well as challenging job demands and decrease hindering job demands. While increasing structural or social job resources refers to behaviors such as feedback-seeking and developing one’s own capabilities, decreasing hindering job demands is targeted at making work less mentally and emotionally exhausting. Scholars have found that crafting in terms of job resources and demands turns out favorable for employee well-being (e.g., Bakker et al., 2012) also on the daily level (Petrou et al., 2012).

Taken together, those previous job crafting approaches define job crafting solely in terms of the characteristics of the job such as making changes to tasks and relationships at work (Wrzesniewski and Dutton, 2001) or in terms of job demands and job resources (Tims et al., 2012). Yet, those studies are relatively mute about how job crafting is related to contextual aspects such as the time and spatial dimensions of work. In today’s new world of work, knowledge workers are able to execute their work activities anywhere anytime, but those practices have led to both positive and negative outcomes for employee well-being, performance, and work-life balance. Hence, it is increasingly important that employees proactively craft changes to the location and timing of work to remain engaged, productive and to retain their work-life balance on a daily level. Thus, the extension that we make is that in the context of time-spatial flexibility, the time and location/place categories become subject to daily job crafting. We call this type of job crafting *time-spatial job crafting* where employees make active changes to their work, relating to working hours, places, and locations of work. Time-spatial job crafting and the previously discussed existing job crafting approaches can co-exist. For instance, employees who came to the conclusion to work from home on a particular day can still change the scope or number of their tasks to derive a different meaning for their work or they can still ask colleagues for advice (increasing social job resources) (e.g., through the use of ICT).

Time-spatial job crafting resonates with the idea that “flexworkers have to assume more responsibility for managing themselves and their whole lives” (Richardson and McKenna, 2014, p. 734) by reordering their lives.

We formally define *time-spatial job crafting* as a context-specific type of job crafting in which employees (a) reflect on specific work tasks and private demands; (b) select workplaces, work locations, and working hours that fit those tasks and private demands; and (c) possibly adapt either their place/location of work and working hours or tasks and private demands to ensure that these still fit to each other thereby optimizing time/spatial-demands fit. This definition is analogous and bears some similarities to the self-regulatory construct of reflexivity, which has been defined at the group level as “the extent to which group members overtly reflect upon, and communicate about the group’s objectives, strategies (e.g., decision making), and processes (e.g., communication), and adapt them to current or anticipated circumstances” (West, 2000, p. 296). Reflexivity is said to consist of three different components, namely reflection, planning, and action (for reviews see Schippers et al., 2014), which represent an iterative cycle of reflection, planning, and action (Schippers et al., 2017). Similarly, we suggest that time-spatial job crafting also consists of three different components, namely reflection, selection, and adaptation that can be presented in a chain of reflection, selection and if necessary, adaptation. Please refer to Figure 1.

### Components of Time-Spatial Job Crafting

#### Reflection

Reflection at the individual level is usually understood in terms of a learning process among individuals in which they examine their past behavior and assess its contribution to performance (for a review, see Ellis et al., 2014). According to Schön (1983), reflection represents serious consideration of past actions and experiences with the aim to evaluate them for future actions. Indeed, reflection in the organizational learning literature is recognized as one central element in learning (Moon, 1999; Høyrup, 2004). Applying this to the context of time-spatial flexibility, reflection can be regarded as a deliberate process of thinking about the tasks and private demands and working hours, places, and locations of work available on any particular day. While considering all the different alternatives, employees may use past experiences to evaluate workplace options for their current choice. They may think about their past workplace/work location and working hour choice and reflect on the benefits/drawbacks of this choice.

We argue that especially when it comes to working with time-spatial flexibility, employees often do not reflect on optimization issues, and often routinely opt for the same workplace (Wessels, 2017). This leads to a mismatch between their task and/or private demands on the one hand and work locations, working hours, and workplaces on the other hand. If employees reflect carefully, they are more likely to detect potential disadvantages of certain flex arrangements for task or private demands.

There is indeed evidence that reflection increases awareness in a variety of contexts – for example, students’ self-awareness of their personal learning style (Kanthan and Senger, 2011), knowledge of mental mistakes (Kahneman, 2011), and awareness of biases and errors (Schippers et al., 2014). Building on this literature, we propose that reflection on task and private demands is likely to foster awareness of the requirements of a particular workday and sensitize employees to the nature of each workplace, work location, and working hours. As such, reflection constitutes the cognitive component of time-spatial job crafting. Once employees have reflected, they can more readily engage in selection, which constitutes the behavioral component.

#### Selection

Selection can be understood here as the actual choice of working hours, work locations, and workplaces, which is then likely to play a part in reaching the best time/spatial-demands fit. The actual choice of a workplace, work location or working hour is the result of the conscious consideration of and choice between alternatives (cf. Vohs et al., 2008). In such a reflective system (Strack and Deutsch, 2004), selection is the outcome of reasoning leading to the choice about the viability of a given action, which is in our case the selection of the right workplace, location or working hour (cf. Ajzen, 1991; Bandura, 1997). Selection may be equal to action in the reflexivity literature, which is defined as “goal-directed behaviors relevant to achieving the desired changes in team objectives, strategies, processes, organizations or environments identified by the team during the stage of reflection” (West, 2000, p. 6). Action is seen as a means to try out assumptions by practical experience (for a review see Widmer et al., 2009).

While reflection and selection may work quite well for days that are fairly predictable, for instance, the decision to work from home when one needs to pick up the kids from school, not all days are equally plannable and may also have unforeseen demands. Therefore, time-spatial job crafting also includes an element of adaptation, which increases in importance when employees are working from a workplace inside the central office.

#### Adaptation

Sometimes employees may face hindrances that prevent them from executing their work tasks in their desired place/location or during the desired time and also perceive problems and/or constraints that may disable them to make the best timing or location decision. Indeed, job crafting may be a more enduring process that can contain adjustments and change, which result from the perceived challenges that limit the opportunities for job crafting (Berg et al., 2010). On the individual level, adapting refers to “performing adaptive behaviors that address changing conditions” (Hirschi et al., 2015, p. 1) and we propose that behaviors such as either changing the workplace, work location or working hours or changing particular tasks/private demands denote illustrations of adapting within the time-spatial job crafting construct.

Key of adaptation in time-spatial job crafting is that timing/location or tasks choices may be adapted in hindsight. Various circumstances may require adaptation. First, it is often the case that employees only realize in hindsight that they made the wrong choice in terms of the time/spatial-demands fit. For instance, even though employees might know that they actually need to work in silence, they could still decide to work in the open office space in order to sit next to a particular colleague they have not seen for a while. Second, depending on the occupancy rate, the reverse situation is also possible. For instance, by means of reflection, employees may conclude that they need a high level of concentration. If the only workplace that is free within the open office space and commuting back home is not an option, employees may choose to engage in a different task that requires less concentration and silence. Third, most workdays involve multiple activities that cannot be readily foreseen in the morning but which may require several different types of workplaces. Therefore, employees also need to adapt where they work to make sure that the workplaces are appropriate to the task at hand. This also suggests that employees need to be able to adjust their work situation “on the fly”; thus, having mini chains of reflection/selection/adaptation each day. In Table 1, we exemplified time-spatial job crafting behavior according to the three dimensions. Overall, we propose that:

**Table 1:** Examples of time-spatial job crafting.

Form	Example reflection	Example selection	Example adaptation
Time-job crafting-Tasks and Private demands	Underlying questions: What do I need to do today? - I need to finish a paper, write emails, and have two meetings with colleagues What are my private demands for today? - I need to bring my kids to school Specific questions: Which working times do I have available for my tasks and private demands? - My day today begins at 6 AM and ends 10 PM; standard office hours are from 8 AM to 5 PM, but I can also work before or after that - I need to bring my kids to school before 9 AM - I have a meeting at 3 PM with my colleagues	- I choose to start working after I will have brought my kids to school - I will work on the paper I need to finish in the morning because I am most productive in the morning - I will write emails in the afternoon	- I need to finish answering my emails in the evening because I did not finish writing my paper in the morning and used the time in the afternoon for my paper
Spatial-job crafting-Tasks and Private demands	Underlying questions: What do I need to do today? - I need to finish a paper, write emails, and have two meetings with colleagues What are my private demands for today? - I need to bring my kids to school Specific questions: Which working locations/workplaces do I have available for my tasks and private demands? - I can work from home, on the go and from the different office spaces inside the office	- I decide to work from home in the morning since I need to work in piece in quiet to finish my paper - I drive to the office after lunch because I have a meeting at 3 PM with colleagues - I decide to work in the open office space so that I can sit close to my colleagues and also because a closed office space was not available to continue working on that paper	- I switched my office place to a closed office space because it was hard for me to concentrate on the paper in the open office space


#### Proposition 2

Time-spatial job crafting consists of a cognitive component, namely reflection, and two behavioral components, namely selection and adaptation.

### Consequences of Time-Spatial Job Crafting

As suggested above, time-spatial job crafting is essential in optimizing time/spatial-demands fit. While time-spatial flexibility can have both desirable and undesirable consequences for well-being, performance, and work-life balance due a time/spatial-demands fit or misfit, we argue that the extent to which this occurs may be contingent upon time-spatial job crafting. Whether employees experience an environment as beneficial or detrimental depends on their requirements of a particular workday. Time-spatial job crafting is likely to help employees realize these resulting in an optimal time/spatial-demands fit.

For instance, on a particular workday, employees may come to the conclusion that they need to engage in focused work and that they will not require a high level of support from colleagues or supervisors (time-spatial job crafting in terms of the task) and that they need to pick up their children from school at 4 PM (time-spatial job crafting in terms of private demands). Once they have reached that conclusion, they are more likely to choose to work in a silent room or from home rather than in an open office space (selection). This would result in the best time/spatial-demands fit for this particular day augmenting work outcomes. When employees are able to seek out work locations, workplaces, and working hours that fit their private and task needs, they are more likely to invest their capabilities fully at work and this should give them more energy and should make them more productive and result in a greater work-life balance. Hence, by modifying time and spatial aspects of the job so that these fit employee’s own task and private demands, they are likely to boost their own engagement, performance, and work-life balance. Prior research has indeed shown that job crafting behavior is linked to higher work engagement (e.g., Petrou et al., 2012; Rudolph et al., 2017). Hence, we suggest:

#### Proposition 3

Time-spatial job crafting leads to a time/spatial-demands fit, thereby leading to higher work outcomes.

Next to having positive effects on work outcomes, we also propose that engaging in time-spatial job crafting behavior leads to a higher person-job fit. As mentioned earlier, the fit between an employee’s competencies such as personal needs and abilities and the requirements of the job is denoted as person-job fit (Kristof-Brown et al., 2005). The notion behind person-job fit is that fit is likely to occur if the employee possesses the necessary skills to meet the requirements of the job (Kristof-Brown et al., 2005). Edwards (1991) distinguished here between demands-abilities fit, in which employees’ knowledge, skills, and abilities (KSA) match with the requirements of the job and needs-supplies fit to refer to the situation an employee’s needs are met by the organization. Applying this to context of time-spatial flexibility implies that employees need to have certain skills, knowledge, and abilities in order to work successfully when granted flexibility. Those skills encompass reflecting, selecting, and adapting so that they are able to match working hours, work locations, and workplaces to personal and task demands. Hence, we propose that time-spatial job crafting can be regarded as a necessary tool when granted flexibility that helps to achieve a good person-job fit, which, in turn may lead to higher levels of organizational commitment (Kristof-Brown et al., 2005).

#### Proposition 4

Time-spatial job crafting leads to greater person-job fit and to higher levels of organizational commitment.

### Antecedents of Time-Spatial Job Crafting

As engaging in time-spatial job crafting seems to be critical in the new world of work, this raises the question what triggers employees to do so. The willingness to engage in time-spatial job crafting is likely to depend on various individual and organizational characteristics at the trait level. On the individual level, if employees have a negative attitude regarding time-spatial flexibility it seems unlikely that they will use time-spatial job crafting to make optimal use of time-spatial flexibility. Attitudes are understood as favorable or unfavorable judgments regarding objects, people, or events (Bohner and Dickel, 2011; Robbins and Judge, 2014), hence we understand a positive attitude toward time-spatial flexibility as employee’s favorable judgments about the practice. This involves for instance seeing the benefits of time-spatial flexibility in terms of places as activity specific spaces, which help to accomplish tasks more efficiently. With regard to time-flexibility, adjusting working hours in a flexible manner also needs to be regarded as valuable for one’s work in order for employees to engage in time-spatial job crafting. If employees do not see these benefits, it is highly unlikely that they will start optimizing their work environment.

#### Proposition 5a

A positive attitude toward time-spatial flexibility leads to time-spatial job crafting.

It seems that time-spatial job crafting and boundary management have some overlap. According to boundary management theory, individuals manage boundaries to organize certain domains in their life (Ashforth et al., 2000). The term ‘boundary work’ was coined by Nippert-Eng (1996) to refer to how individuals build, dismantle, and maintain the work-home border. Managing those work-home boundaries takes place on a continuum ranging from boundaries that are permeable and highly integrative to impermeable and segmented (Ashforth et al., 2000). Whether boundaries are integrated, separated, or alienated depends on individual’s preferences. Boundary management theory is concerned with all domains of work and thus broader than the concept of time-spatial job crafting. Time-spatial job crafting is highly context specific as it only relates to knowledge work organizations that offer time-spatial flexibility.

However, time-spatial job crafting does not solely explicitly relate to solving or negotiating conflicts between work and family life. On the one hand, the time-spatial job crafting model suggested that employees who have time-spatial flexibility need to find a fit between time and space and their tasks. This solely concerns negotiating demands with respect to their work role for which time-spatial job crafting is likely to help. Hence, this is not about managing work-home boundaries but about managing tasks and work locations, working hours, and workplaces. On the other hand, the time-spatial job crafting model is also concerned with optimizing the fit between time and space and private demands. With this dimension, there might be some overlap between boundary management since time-spatial job crafting related to private demands is also concerned with managing demands between work and home. However, the literature on boundary management is silent with respect to the underlying processes that lead to the decision regarding when and where to work. While Kreiner et al. (2009) do shed light on how people manage boundaries by introducing four types of boundary work tactics at the behavioral, temporal, physical, and communicative level to negotiate demands between work and home, they do not explain the underlying processes that lead to for instance to adapting physical boundaries. This more flexible perspective is offered by our theorizing on time-spatial job crafting and the accompanying process of reflection, selection, and adaptation.

Boundary management style however can be an important antecedent of time-spatial job crafting related to private demands and hence, we introduce boundary management style as a possible antecedent of time-spatial job crafting related to private demands in the model, however, we argue only for time-spatial job crafting in terms of private demands. Boundary management style refers to “a general approach an individual uses to demarcate boundaries and regulate attending to work and family roles” (Kossek and Lautsch, 2012, p. 155). Individuals thereby make use of different boundary management styles to manage those boundaries. Kossek and Lautsch (2012) proposed that next to the separation-integration continuum, where individuals either separate or integrate work and family, individuals can also adopt a more hybrid approach alternating between separation and integration. The extent to which employees employ either of these styles depends on their boundary-crossing preferences and their work-family role identity centrality (Kossek and Lautsch, 2012). While segmented boundaries result in a higher inflexibility and in a more rigid separation of roles in terms of times and place, integrated boundaries foster greater integration of roles.

We argue that an employee’s preference for integration, separation or alienation (which will also depend on the preference of the family) will influence time-spatial job crafting in terms of reflecting and choosing where and when to work aligned with private demands. A preference for a particular boundary management style will help employees in reflecting about the difference options available and will ultimately result in selection of a specific work location which is in line with the employee’s boundary management style. For instance, employees who prefer to separate home and work in a strict manner, are more likely to come to the conclusion that it is not advisable for them to work from home when kids are around (reflection) and thus choose their timing of work (selection) in such a manner that it does not interfere with family responsibilities (e.g., going to the office earlier, finishing un-finished tasks the next day).

#### Proposition 5b

An employee’s boundary style preference for integration, separation or alienation will positively influence time-spatial job crafting in terms of private demands.

Furthermore, at the individual level, individual needs for autonomy, competence, and relatedness (Deci and Ryan, 1985) may play a crucial role in shaping time-spatial job crafting behavior. Bindl et al. (2018) showed in their extended framework of job crafting that indeed individual needs are decisive for engaging in job crafting behavior. They found that employees who had a stronger need for autonomy, relatedness and competence were more likely to engage in task crafting, skill crafting, and relationship crafting, respectively. In a similar vein, we propose that the need for autonomy may be crucial for time-spatial job crafting since such individuals have a desire to exercise control over their actions (Deci and Ryan, 1985). Since deliberately thinking and choosing where and when to work can be understood as such a control-taking process, the need for autonomy might trigger time-spatial job crafting. The need for relatedness describes an individual’s desire to feel connected to others (Deci and Ryan, 1985). An individual who has a strong need for relatedness may be more likely to reflect on when and where to work since they want to stay close to colleagues and their choice may be (partly) contingent on colleagues. Finally, the need for competence, which represents an individual’s desire to feel skillful in one’s behavior (Deci and Ryan, 1985), may also stir time-spatial job crafting behavior, since time-spatial job crafting represents a way of how individuals can better handle time-spatial flexibility. Hence, we propose:

#### Proposition 5c

Employee’s needs for autonomy, competence, and relatedness leads to time-spatial job crafting.

At the organizational level, perceived organizational support may also play a role. If employees perceive that flexible working is not accepted within the organization, or fear negative consequences for their career, it seems unlikely that they will use time-spatial job crafting to make optimal use of time-spatial flexibility. Research at Microsoft Netherlands, which moved toward new ways of working, has shown that it is indeed important that the whole organization including the CEO of the company approves of this change process (van Heck et al., 2012). If an employee realizes that fellow colleagues do not appreciate him or her working flexible, it is highly unlikely that this employee will engage in time-spatial job crafting to make the most out of time-spatial flexibility. That is indeed what Fursman and Zodgekar (2009) found in their study. Employees reported as one barrier to make use of time-spatial flexibility a non-supporting organization. Likewise, if an employee recognizes that flexible working has detrimental effects on his or her career, it is also not very likely that he or she will become a time-spatial job crafter. Prior research has shown that employees are less inclined to make use of time-spatial flexibility when they fear negative consequences for their career (Fursman and Zodgekar, 2009). Furthermore, prior research on job crafting has also shown the prominent role of the leader as well as social support from colleagues in fostering job crafting behavior (Harju et al., 2018). We also argue that in order for employees to engage in time-spatial job crafting, a supportive leader and colleagues may trigger employees to engage in time-spatial job crafting. Taken together we suggest that.

#### Proposition 5d

Employees are more likely to engage in time-spatial job crafting when they perceive that the organization and co-workers accept time-spatial flexibility and when they do not fear negative consequences for their career.

#### Proposition 5e

Employees are more likely to engage in time-spatial job crafting when the experience support from the leader and from colleagues.

### Intricacies to Time-Spatial Job Crafting

While the preceding discussion suggests that reflecting on and selecting workplaces, work locations, and work hours is straightforward, in fact, employees may also be likely to resist reflecting since conscious reflection may be something that employees are often not familiar with and may elicit defense reactions. Hence, since time-spatial job crafting is a behavior that needs to be learned, resistance to reflect (i.e., Piderit, 2000) may hinder to optimize a time/spatial-demands fit and lead to positive work outcomes in the short-term.

Also, on any given workday employees may face conflicting demands that make the selection of the right workplace or working hours more difficult. Making choices turns out to be more troublesome at whatever point various needs, objective or values, are in conflict (Brandstätter et al., 2006). Conflicting demands either within the work domain or between the work and home domain can create what is commonly termed role conflict within the same role (intra-role conflict, or between two roles (inter-role conflict; Kahn et al., 1964), which occurs “when the behaviors expected of an individual are inconsistent (Rizzo et al., 1970, p. 151). For instance, even though employees would perhaps like to work from home so that they can work in perfect silence, at the same time they also might have several meetings that require them to be at the main office. Even if employees consciously decide to work from home, unlearning to resist going to the fridge, lying on bed or watching TV (Howgego, 2019), hence to procrastinate, can take some effort and time.

Also, the choice over when and where to work may depend on the choices of colleagues. Evidence suggests that employees base their workplace/work location choice on the decision of their colleagues (Rockmann and Pratt, 2015), which may not be in line with private or task demands. Hence, managing those opposing demands is difficult and creates extra effort; effort in the form of more reflection, selection, and potentially adaptation. Thus, time-spatial job crafting can be a strenuous activity in itself, although one would also expect that over time “practice makes perfect,” and choices can be made with less effort. Consequently, it is likely that the proposed benefits of time-spatial job crafting will be less strong in the short run and increase in the long term (cf. Schippers et al., 2013).

#### Proposition 6

Time-spatial job crafting could be an energy-draining activity in itself, and therefore, the positive role of time-spatial job crafting will be more positive in the long-term than in the short-term.

## Discussion

In this paper, we have explored the implications of time-spatial flexibility for work outcomes and person-job fit on a daily level. We have applied proactive work design literature and literature on flexible working practices to explain that individuals can make choices over workplaces, work locations, and working hours that enable them to either exploit the advantages of time-spatial flexibility or run the risk of being affected by the disadvantages. In order for employees to make the best choice in terms of their tasks and private demands, we introduced the concept of time-spatial job crafting as a context-specific type of job crafting. We proposed that employees may use time-spatial job crafting as a technique that allows them to reap the benefits of time-spatial flexibility and avoid its drawbacks to optimize time/spatial-demands fit on a day-to-day basis.

### Implications for Job Crafting and Flexibility Research

Theoretically, the model extends current theorizing efforts in flexibility research. Previous literature has shown that flexibility can have both – a positive and negative effect at the cross-sectional level; through time/spatial-demands fit and time-spatial job crafting, we explained when flexibility will be positive and negative on the daily level. Hence, we incorporated the role of time into flexibility research helping to explain some of the ambiguous outcomes. In addition, we extend the job crafting literature to the context of flexibility. Whereas the traditional job crafting literature construes job crafting in terms of job characteristics (Wrzesniewski and Dutton, 2001; Tims et al., 2012), we postulated that other aspects of the job can also be subject to job crafting and this becomes especially important when working flexibly. Time-spatial job crafting is offered as a tool that should help employees to exploit time-spatial flexibility and that can be regarded as an optimization strategy for using various workplaces, work locations, and working hours, which leads to finding a time/spatial-demands fit. With the term ‘time/spatial-demands fit’ we created a new form of fit, which has never been made explicit by prior flexibility research.

As much as job crafting represents a valuable tool for older workers to find a good person-job fit (Wong and Tetrick, 2017), the suggested positive role of time-spatial job crafting should enable employees to better deal with flexibility and should have a positive impact on work outcomes through a time/spatial-demands fit. We stressed throughout the paper that employees need to become proactive if they want to reap the benefits of time-spatial flexibility.

This paper therefore highlighted the importance of bottom-up approaches to work design in the new world of work. On top of that, we also add to the P-E fit literature by explaining how time-spatial job crafting relates to a better person-job fit.

### Implications for Practice

As HR managers are constantly assessing how different workplace settings may influence performance (Okhuysen et al., 2013), the insights we provide should give them a greater understanding of how work-settings change the nature of work and consequently influence human behavior. Demonstrating the importance of time-spatial job crafting to ensure that employees are able to use various workplaces, work locations, and working hours optimally could become a crucial aspect of managers’ agenda. By making employees aware of how they can make changes within their environment if they reflect on what is needed, managers can show employees how they themselves can increase their own well-being, performance, and work-life balance. This can be achieved, for instance, through a time-spatial job crafting intervention, in which they learn what they themselves can do to enjoy working in such an environment. Several recent studies among various groups of employees have shown that job crafting interventions and trainings can be successful (e.g., Van den Heuvel et al., 2015; Van Wingerden et al., 2017). Such a training might also be of particular importance since there may be cases in which a poor or suboptimal time-spatial choice is not perceived or recognized as such by employees or employees might not be aware that much more effective practices are available and could be used. Awareness could be enhanced via trainings, however, only a continuous assessment of one’s own behavior by employees themselves, managers or also fellow colleagues helps to optimize time/spatial-demands fit over time. On top of that, it is important that employees experience the benefits of time-spatial flexibility and time-spatial job crafting first hand so that they are motivated to engage in time-spatial job crafting as attitudes form directly as a result of experience or social norms (Bohner and Dickel, 2011). This is also important in light of resisting to reflect. Piderit (2000) termed resistance to change as a negative attitude toward a certain change process. Schippers et al. (2014) pinpointed to the hindrance and facilitating factors of reflection (reflexivity) and to trainings and interventions in regard to the latter. Therefore, since time-spatial job crafting is a behavior that needs to be learned, it is important that employees experience the benefits of reflection and learn this in trainings.

### Limitations and Research Agenda

The time-spatial job crafting perspective on work outcomes affords several valuable research opportunities. First of all, researchers interested in flexibility and job crafting should empirically address the model we proposed also in non-Western organizations. Second, it would be interesting to use an intervention study to test the concept of time-spatial job crafting or a case-study and conduct interviews to evaluate the effectiveness of such an intervention, also at the team-level.

Also, time-spatial job crafting imposes interesting challenges for leadership and cooperation. If employees are allowed to engage in time-spatial job crafting, and every employee adjusts time and location choices to his or her own preference, this requires on the one hand increased coordination among employees but also challenges for leadership. Interesting leadership questions the model might provoke are: Is there a preferred leadership style for time-spatial job crafting? How can a leader facilitate employees to engage in time-spatial job crafting? What does time-spatial job crafting mean for leader-membership exchange? It is also interesting in itself to know how to foster good time-spatial job crafting and for whom it may work best. For instance, interesting to investigate in a quantitative study might be whether there exist generational differences in time-spatial job crafting behavior. One might assume that it is easier for generation Y to embrace time-spatial job crafting since they are “pragmatic, open-minded, (…), innovation-oriented, [and] eager to experiment with new solutions” (Sujansky and Ferri-Reed, 2009, p. 135). Longitudinal studies should also address the long-term consequences of time-spatial job crafting. This is important to investigate as we indicated at the start of the article, that we restricted our suggestions to organizations that offer employees time-spatial flexibility. Also, it is conceivable that once employees become used to working in a flexible manner and where the task structure stays stable, time-spatial job crafting can also become a more routine-based behavior (cf. Schippers et al., 2014). This may be an interesting notion for future research to see whether time-spatial job crafting can positively contribute to work engagement, performance, and work-life balance above and beyond its daily effects.

An important caveat to the concept of activity magnet areas in general is that there are certain tasks, such as writing emails or correcting documents that could technically be undertaken from many different workplaces. Where this actually takes place will depend on personal preferences. While some employees prefer to answer an email in private, other workers do not mind doing so within the open office space. A possible avenue for future research may be to explore the role of personal preferences in choices regarding workplaces and working hours. The empirical distinctiveness of time-spatial job crafting and boundary management tactics represents also an interesting avenue for further research.

## Conclusion

In the last two decades, time-spatial flexibility has become a popular approach to redesigning work. A considerable literature emerged to examine the relationship between time-spatial flexibility and various outcomes, amongst other well-being, performance, and work-life balance. However, previous research failed to demonstrate an unequivocal business case for time-spatial flexibility identifying both positive and negative effects on well-being, performance, and work-life balance. We proposed a model of time-spatial job crafting that may help explain why prior studies found diverging and contradicting results. We posited that in order for employees to profit from time-spatial flexibility, time-spatial job crafting – a context-specific form of job crafting that entails reflection on time and place– can be seen as a strategy for staying well and being productive because it helps to find a time/spatial-demands fit. Accordingly, we offer a greater understanding of time-spatial flexibility for managers and a new direction for scholars examining new ways of working: time-spatial job crafting ensures that workers reflect in order to optimize time/spatial-demands fit.

## Author Contributions

CW played the primary role in the conception and drafting of the manuscript. CW and MS developed together the idea of time-spatial job crafting. MS played a major role in shaping the manuscript. SS and AB provided the critical revision to the manuscript and contributed to the conceptualization, writing, and editing. PvB and KP revised the work critically for important intellectual content. All authors contributed to all parts of the manuscript, agreed to all aspects of the work, and approved the final version of the manuscript.

## Conflict of Interest Statement

The authors declare that the research was conducted in the absence of any commercial or financial relationships that could be construed as a potential conflict of interest.
